# An Integrated clinical pathway for diagnosis, treatment and care of rare diseases: model, operating procedures, and results of the project TRANSLATE-NAMSE funded by the German Federal Joint Committee

**DOI:** 10.1186/s13023-021-02092-w

**Published:** 2021-11-12

**Authors:** Daniela Choukair, Fabian Hauck, Markus Bettendorf, Heiko Krude, Christoph Klein, Tobias Bäumer, Reinhard Berner, Min Ae Lee-Kirsch, Corinna Grasemann, Peter Burgard, Georg F. Hoffmann

**Affiliations:** 1 Center for Child and Adolescent Medicine and Center for Rare Diseases, University Hospital Heidelberg, 69120 Heidelberg, Germany; 2 Department of Pediatrics, Dr Von Hauner Children’s Hospital, University Hospital and Munich Centre for Rare Diseases, LMU Munich, 80337 Munich, Germany; 3 Institute for Experimental Pediatric Endocrinology, and Berlin Center for Rare Diseases, Charité - Universitätsmedizin Berlin, 13353 Berlin, Germany; 4 Institute of Systems Motorscience and Center for Rare Diseases, University of Lübeck, 23562 Lübeck, Germany; 5 Department of Pediatrics and University Center for Rare Diseases, University Hospital Carl Gustav Carus, Technische Universität Dresden, 01307 Dresden, Germany; 6 Department of Pediatrics, St-Josef Hospital Bochum and and Center for Rare Diseases, Ruhr-University Bochum, 44791 Bochum, Germany

**Keywords:** Clinical pathway, Rare diseases, Diagnostic odyssey, Evidence-based medicine, Case management

## Abstract

**Background:**

Diagnosis, treatment, and care of patients with rare diseases require multidisciplinary cooperation between medical and paramedical specialities and with patients and families. Innovative genetic diagnostics, whole exome and whole genome sequencing (WES, WGS) has enlarged the diagnostic toolkit but also increased the complexity of the endeavour. Structured multidisciplinary clinical pathways (CPW) can guide diagnosis, treatment, and care of patients with rare diseases, link scientific evidence to clinical practice and optimise clinical outcomes whilst maximising clinical efficiency.

**Results:**

In contrast to the common approach of appending disease-specific CPWs to disease-specific guidelines, we suggest a generic CPW manoeuvring the patient along the way of finding the correct diagnosis by applying the best diagnostic strategy into an appropriate system of treatment and care. Available guidelines can be integrated into the generic CPW in the course of its application. The approach also applies to situations where a diagnosis remains unsolved. The backbone of the generic CPW is a set of multidisciplinary structured case conferences projecting and evaluating diagnostic and/or therapeutic steps, enforcing to integrate best scientific evidence with clinical experience. The generic CPW is stated as a flowchart and a checklist which can be used to record and document parsimoniously the structure, process and results of a patient’s pathway, but also as a data model for research. It was applied in a multicentre setting with 587 cases each with a presumptive diagnosis of a rare disease. In 369 cases (62.8%) a diagnosis could be confirmed, and multidisciplinary treatment and/or care was initiated. The median process time from first contact until confirmation of diagnosis by WES was 109 days and much shorter than diagnostic delays reported in the literature. Application of the CPW is illustrated by two case reports.

**Conclusions:**

Our model is a tool to change the diagnostic odyssey into an organised and trackable route. It can also be used to inform patients and families about the stages of their individual route, to update health care providers only partially involved or attending specialised treatment and care, like the patient’s or family’s primary physician, and finally to train novices in the field.

## Background

In Germany approximately four million people are suffering from rare diseases [1]. Many patients endure a long odyssey before their condition can be diagnosed and managed appropriately. Of them, about 80% have a genetically determined disease, often manifesting in (early) childhood. Predominantly, these diseases are clinically heterogeneous, complex and often associated with a chronic and deteriorating course [2]. Therefore, multidisciplinary care of specialists, general practitioners, nurses, therapists, and social workers must be established to provide best care for patients and families [3–5].

In 2009 the Council of the European Union addressed the problem of rare diseases [6]. In 2010 the German Federal Ministry of Health (BMG) together with the German Federal Ministry of Education and Research (BMBF) and the Alliance for Chronic Rare Diseases (ACHSE e.V.) [7] founded the National Action League for People with Rare Diseases (NAMSE) to devise measures for improving health care for patients with rare diseases [8]. Three years later, 2013, the German National Plan of Action for People with Rare Diseases was published [9]. Overall, 52 policy proposals were compiled covering a wide spectrum of tasks on information management, establishment of diagnostic pathways, care-giving structures, and research. However, up to date only few recommendations could be put into action.

To improve the care of patients with rare diseases the German Federal Joint Committee (G-BA) funded the innovation project TRANSLATE-NAMSE from April 2017 until September 2020 [10, 11]. TRANSLATE-NAMSE was a health care project (development and establishment of new structures and processes across different healthcare providers and disciplines) and not a health care research project (scientific investigation of patient-centred and population-based care). Nine German centres for rare diseases (Berlin, Bonn, Dresden, Essen, Hamburg, Heidelberg, Lübeck, Munich, Tübingen), two health insurance companies (AOK Nordost; Barmer GEK) and the Alliance for Chronic Rare Diseases (ACHSE e.V.) established a consortium to design, test and evaluate a model of structured care for patients with rare diseases [12].

TRANSLATE-NAMSE had three sub-projects. Sub-project number one was devoted to make diagnoses in hitherto undiagnosed patients with a long diagnostic odyssey, including structured case-conferences and the use of innovative genetic testing (whole exome sequencing). Sub-project number two had to organize the transition from paediatric to adult care of patients with rare diseases as a structured and quality assured process [13]. Here we present sub-project number three which had the goal to develop a generic clinical pathway (CPW) for confirmatory diagnostics, treatment and care of individuals with a presumptive diagnosis in one of five defined groups of rare diseases, i.e. rare amaemias, endocrinopathies, autoinflammatory diseases, primary immune deficiencies and inborn errors of metabolism. The five disease groups were selected since they include diseases from the German newborn screening panel or the participating centres had acknowledged expertise.

A Cochrane systematic review and meta-analysis [14] defined clinical pathways (CPW) as structured multidisciplinary care plans, which are used by health services to detail essential steps in the care of patients with a specific clinical problem with the aim to link evidence to practice and optimise clinical outcomes whilst maximising clinical efficiency. CPWs may lead to reductions in hospital complications, improved documentation, significant reduction of length of stay and a decrease in hospital costs. Traditionally CPWs translate guidelines for specific disorders into local management protocols where pathway development is described corresponding to guideline development [15]. As a result, disease-specific CPWs are supplementary material to guidelines or later on appended to already existing guidelines [16]. However, as clear CPWs only exist for the most frequent rare diseases, if at all, this approach will not fit with situations where a diagnosis has yet to be established and the disease matching guideline to be determined. Our study pursued two objectives. In phase one a generic CPW should be developed, manoeuvring the patient along the way of finding the correct diagnosis or applying the best diagnostic strategy and guiding the patient into an appropriate system of treatment and care. The CPW should be graphically displayed as a flowchart as well as a checklist explicating the flowchart elements and allowing to document the workflow for single individuals. In phase two the approach should be tested in clinical practice.

## Methods

In phase one the flowchart and the checklist were developed by a CPW development group with representatives of all disciplines involved in diagnosis, treatment and care of the five defined groups of diseases [15]. Available guidelines for the five defined groups of rare diseases were identified, e.g. [17, 18], and current and past practice were reviewed. Development started with the flowchart which was then translated into a checklist describing explicitly the actions in each particular step, also allowing to document results and corresponding process times. Drafts of both documents were reviewed by all participating centres and finalized via a circulation procedure. In phase two the final documents were implemented as PDF forms in the hospital information systems of six university medical centres (Berlin, Dresden, Essen, Heidelberg, Lübeck, Munich) and staff was educated to apply the CPW in clinical practice. WES was performed by four university departments of human genetics (Berlin, Bonn, Munich, Tübingen) which were members of the TRANSLATE-NAMSE consortium. Data in the PDF forms were read out into CSV files, merged across all participating centres, imported into SPSS 26, checked for plausibility and completeness, and analysed descriptively. Results of the clinical application of the CPW were evaluated independently by the Berlin School of Public Health (https://bsph.charite.de/en/) and the Center for Evidence-Based Healthcare, University Hospital Dresden (https://www.uniklinikum-dresden.de/de/das-klinikum/universitaetscentren/zegv/center-for-evidence-based-healthcare/center-for-evidence-based-healthcare).

## Results

### The clinical pathway as a flowchart

**Fig. 1:**
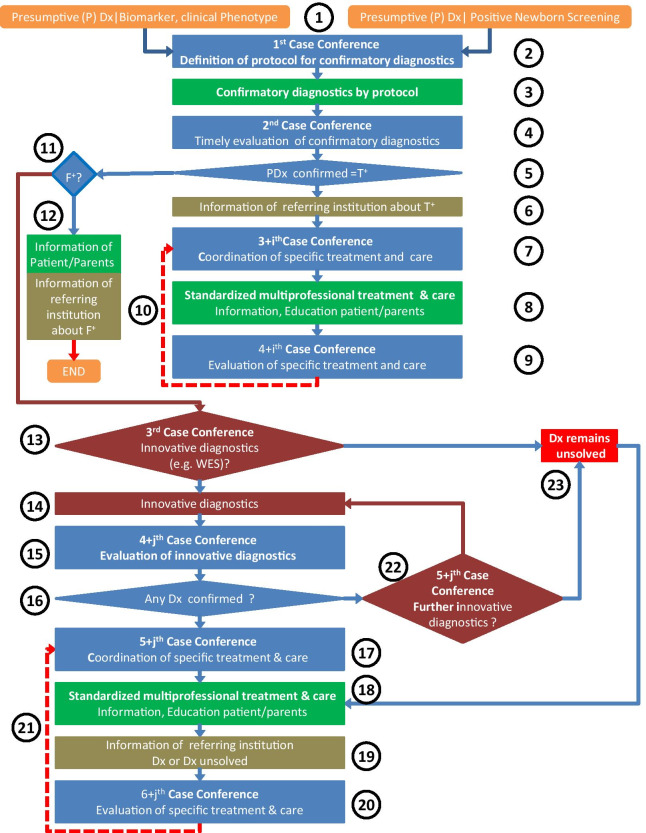
The clinical pathway as a flowchart. Following conventions for flowcharts, rounded rectangles indicate start or end of a process, rectangles represent actions, and rhombuses binary decisions. Vertical arrows starting from rhombuses always point to the next step if the answer is yes, horizontal arrows point to the next step if the answer is no. Circled numbers 1 to 23 refer to the explanation of the particular step in the text. 3 + i with i = 0, 2, 3, ….: in step 7 for i = 0 the third case conference coordinates treatment and care for the first time. When results of the evaluation of treatment and care in step 9 are fed back to step 7, the conference deciding continuation or modification of treatment and care is the 3 + 2 = 5^th^ case conference, and so on. 4 + j with j = 0, 2, 3, 4, …: in step 15 for j = 0 it is the 4^th^ case conference. If in step 16 PDx is not confirmed, and in Step 22 (the 5^th^ conference) it is decided to perform further innovative diagnostics, than returning to step 14, the number of the case conference will be 4 + i = 2, i.e. the 6^th^ conference, and so on. Presumptive (P) Dx|Biomarker, clinical Phenotype: presumptive diagnoses given Biomarker or clinical Phenotype; the symbol (|) is to be read as “in case of”. Dx: Diagnosis, F^+^: false positive, NBS: newborn screening, PDx: presumptive diagnosis, T^+^: true positive, WES: whole exome sequencing

Figure 1 shows the steps of the suggested CPW as a flowchart. The meaning of symbols and abbreviations is explained in the legend. The chart allows to display different courses of events, depending on the results of diagnostic procedures and/or expert clinical decision. The step numbers in the text refer to the corresponding step of the flowchart. Concrete actions in the different steps of the generic CPW are directed by structured interdisciplinary case conferences enforcing integration of best scientific evidence with clinical experience [19]. Participants and disciplines in case conferences are recruited by a medical coordinator according to available guidelines and/or clinical requirements. The process either starts from a positive newborn screening (NBS) result or from a clinical phenotype and/or one or more biomarkers (step 1). In a first case conference (step 2) either a standardised protocol for confirmatory diagnostics is triggered (if the presumptive diagnosis comes out from a defined screening panel) or an ad hoc protocol for confirmatory diagnostics is established based on available information (biomarkers and/or the clinical phenotype). Results of all confirmatory diagnostics (step 3) must be timely evaluated in a second case conference (step 4), resulting in the decision whether the presumptive diagnosis (PDx) was confirmed or not (step 5).

If a PDx was confirmed the institution which referred the patient has to be informed about the result (T^+^) (step 6). Disease-specific treatment and care (including information and education of patients and/or parents) is coordinated in a third case conference (step 7), timely implemented (step 8), and evaluated after an appropriate time in a fourth case conference (step 9). A feedback loop (step 10) from step 9 to step 7 secures long-term monitoring (decisions about continuation or modification) of treatment and care.

If the PDx could not be confirmed in step 5, it must be decided whether the case must be evaluated as false positive (F^+^) (step 11). If this is the case, patients and/or parents as well as the referring institution are informed about the results (step 12) and the clinical pathway ends here.

If the PDx could neither be confirmed nor dismissed as false positive, the clinical pathway moves to an additional case conference (step 13) with the aim to decide whether further, particularly innovative diagnostics, e.g. whole exome sequencing (WES), whole genome sequencing (WGS) should be accomplished. Following a positive decision, innovative diagnostics is performed (step 14) and evaluated (step 15). If a diagnosis can be made (step 16), disease-specific treatment and care (including information and education of patients and/or parents) is coordinated in a further case conference (step 17), implemented (step 18), and the referring institution is informed about the diagnostic result and decisions about treatment and care. After an appropriate period of time treatment and care are evaluated in an additional case conference (step 20). Results of the evaluation enter a feedback loop (step 21) allowing long-term monitoring and adaptation of treatment and care.

If a diagnosis cannot be reached by step 16 of the clinical pathway, a case conference (step 22) decides whether further innovative diagnostics should be executed. The circuit of steps 22, 14, 15, 16, 22 may run through several times, always in the setting of a multidisciplinary case conference. In the same way as in step 13 it can be decided in step 22 that innovative diagnostics will not be useful, further investigations should be stopped, and that the diagnosis has to remain unsolved (step 23). If the diagnosis is confirmed by innovative diagnostics, disease-specific treatment and care (including information and education of patients and/or parents) is coordinated in a 5 + j^th^ case conference (step 17) and timely implemented (step 18). The referring institution is informed about diagnostic results and therapeutic decisions (step 19). Treatment and care are evaluated after an appropriate time in an additional case conference (step 20). A feedback loop (step 21) from step 19 to step 17 allows long-term monitoring (decisions about continuation or modification) of treatment and care and the outcome of the patient. Supposed the case conference in step 13 decides that innovative diagnostics is not promising, the diagnosis remains unsolved (step 23), and the patient is referred to symptomatic care (step 18) and monitoring (steps 20, 21). The sequential organisation of the flowchart does not exclude parallel actions. For example, as delineated in the description of case 2 below, even if the final diagnosis is not yet established and innovative diagnostics is decided to be done (step 13), symptomatic patients as well as asymptomatic patients at risk for clinical onset of disease can get standardised prophylaxis, treatment, care and monitoring (steps 7, 8, 9, 10).

### The clinical pathway as a checklist

**Fig. 2:**
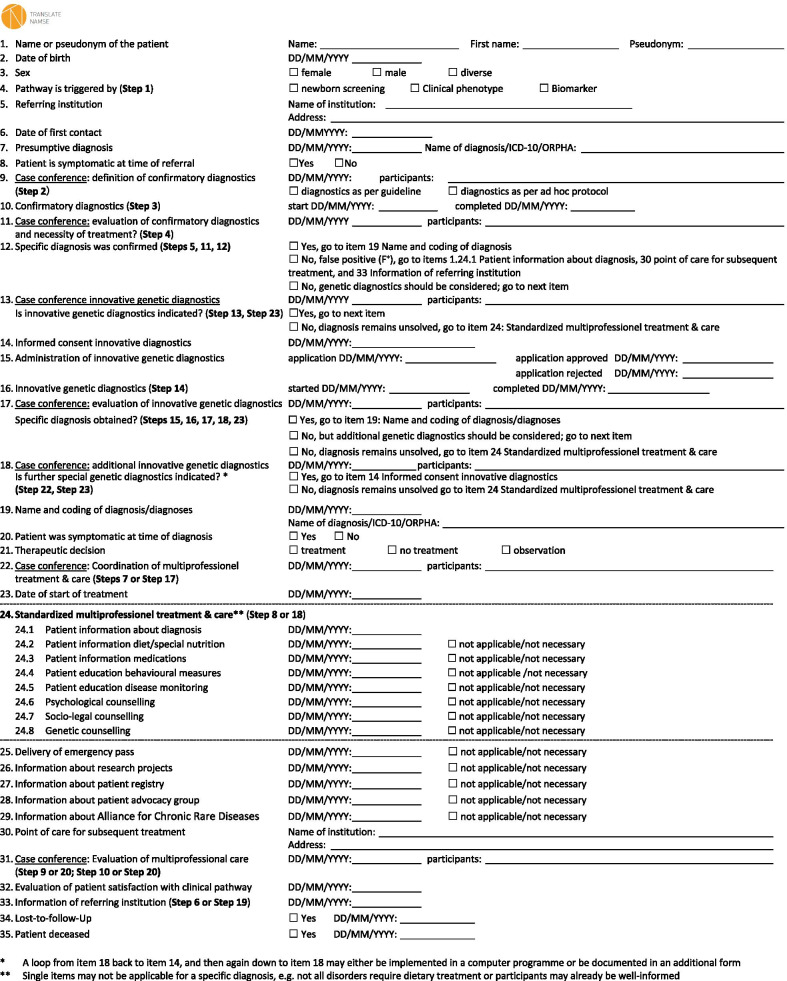
The Clinical Pathway as a Checklist (Step numbers refer to the steps in Fig. 1)

Figure 2 shows the transformation of the steps of the flowchart in Fig. 1 into the items of a checklist. This checklist allows to document the CPW of a patient in a parsimonious but comprehensive way. Single process steps are now fleshed out by content, i.e. the items define the required type of action or information and replies to the items describing what essentially has been done. Multidisciplinary compositions of case conferences, as well as process times can be documented. There are two reasons why the numbers of the steps in the flowchart do not correspond to the numbers of the items in the checklist. First, the checklist is more comprehensive than the flowchart, and second the flowchart is not linear but is branching out depending on the result in a decision rhomb. For example, if in step 5 the presumed diagnosis is not confirmed and classified as false positive (step 11), patients and the referring institution are informed about all results. In the checklist this is documented in Item 12 from where the user is directly forwarded to items 24.1 (information of the patient about diagnosis) and 32 (information of the referring institution). The eight actions provided in item 24 (standardized multiprofessional treatment and care) can by selected according to the medical requirements of the particular diagnosis and individual needs of the patient.

### Two case reports illustrating the clinical pathway model

To illustrate the application of our clinical pathway model, we describe a patient coming with a positive NBS result of slightly elevated 17-hydroxyprogesterone in dried blood spot (case 1) and a patient presenting postpartum with a clinical phenotype of ambiguous genitals (case 2).

**Case 1:** male, born June 2019; presumptive diagnosis based on positive newborn screening result. Step numbers in brackets refer to the corresponding step in the flowchart in figure 1.

After uneventful pregnancy a full-term male infant was born spontaneously. Parents are unrelated healthy Caucasians. Newborn screening at day 3 postpartum (pp) revealed slightly elevated 17-hydroxyprogesterone (17-OHP) in dried blood spot (69 nmol/l, cut-off < 50) [20]. Screening recall according to the German screening guidelines revealed elevated 17-OHP in blood dried spot (82 nmol/l, cut-off < 30) at day 10 pp while the newborn remained asymptomatic. Determination of serum 17-OHP was initiated (step 1).

In a first case conference, organized by the medical coordinator of the centre for rare diseases Heidelberg, a paediatric endocrinologist together with the guarantor for medical validation of the newborn screening results met. Based on the now clearly elevated 17-OHP it was decided to follow-up the presumptive diagnosis of 21‐hydroxylase deficiency (21-OHD). Concordant with guidelines [21] immediate hospitalization for initiating the confirmation diagnostics as well as start of the treatment was recommended (step 2).

On admission a 15-day old dystrophic male presented with icteric skin colour, male infantile genitals with testes volumes of 1 ml each and no hyperpigmentation. Serum 17-OHP (4318 ng/dl) and 21-desoxycortisol (21DF) (1175 ng/dl) were significantly elevated, suggesting classical congenital adrenal hyperplasia (CAH). Additional laboratory examinations revealed sodium 133 mmol/L (norm 131–145), potassium 5.45 mmol/L (3.6–6), glucose 91 mg/dl (60–100), total bilirubin 15.9 mg/dl (< 3); direct bilirubin 1.3 mg/dl (< 0.3); normal blood gas analysis. Determination of adrenocorticotropic hormone (ACTH), plasma renin activity, 17-OHP and 21DF was initiated. Prompt treatment with a bolus of hydrocortisone 30 mg/m^2^ i.v. was followed by continuous infusion of hydrocortisone 15 mg/m^2^/d. Five days after admission (day 20 pp) the results from the initial serum samples were elevated for serum 17-OHP (6611 ng/dl, N < 300) and 21DF (1581 ng/dl, 2–15), also for ACTH 182 pg/ml (10–50), and plasma-renin-activity 62 ng/Al/h (4–35) (step 3).

In a second case conference (step 4), organized by the medical coordinator of the centre for rare diseases Heidelberg, the paediatric endocrinologist and the guarantor for medical validation of the steroid hormone laboratory confirmed the suspected diagnosis of CAH due to 21OHD (step 5), and recommended additional molecular genetic analysis of *CYP 21A2* (step 3). The institution which referred the patient was informed about the true positive result (T^+^) of the confirmatory diagnostics (step 6).

Meanwhile the i.v. hydrocortisone therapy was substituted for hydrocortisone per os (p.o.), fludrocortisone, and sodium chloride: Alkindi® 1–1-1 mg (15 mg/m^2^/d hydrocortisone granules), Astonin H® 2 × 0.05 mg tablets, sodium chloride solution 10% 5 times 2 ml per day. The next day (day 21 pp) the medical coordinator of the centre for rare diseases Heidelberg invited specialists from paediatric endocrinology, genetics, paediatric radiology, paediatric psychology, and social work for a third case conference to coordinate multidisciplinary care (step 7).

A paediatric endocrinologist informed parents about the diagnosis CAH and the necessity of lifelong treatment and monitoring and trained them how to prevent adrenal crises by increasing glucocorticoids during intercurrent illness (step 8).

A glucocorticoid injection kit for emergency use was demonstrated and prescribed, and a personalised emergency pass was handed out (step 8).

The parents were informed about the German patient organization for CAH [22] and invited to take part in the German register of CAH AQUAPE-AGS [23] and in local clinical studies. An experienced paediatric psychologist offered counselling and support to the family. A social worker informed about legal health issues for patients with a chronic disease (step 8).

At day 19 pp, the patient was discharged, and close outpatient monitoring was initiated. After 5 days of treatment ACTH 10 pg/ml (norm 10–50), plasma-renin-activity 6.8 ng/Al/h (4–35), and serum 17OHP 24 ng/dl (< 300) had normalized, only 21DF remained slightly elevated with 45 ng/dl (2–15).

Four weeks after discharge, molecular genetic results were available revealing a compound heterozygous genotype in the *CYP21A2* gene (step 4). A splice site mutation in intron 2 on one allele completely diminished the enzyme activity. A point mutation in exon 4 of the second allele may result in a residual enzyme activity of 2–4%. The parents, both being carriers, received detailed genetic counselling (step 8).

Six weeks after hospitalization (day 65 pp), the members of the third case conference met again for a fourth case conference (step 9). Based on a checklist established in the project TRANSLATE-NAMSE, the whole process of diagnosis and multidisciplinary treatment and care was carefully evaluated (step 9).

The patient and his family are still followed by the paediatric outpatient clinic Heidelberg, At the age of 2 years mental and motor development are appropriate for age (step 10).

**Comment:** As recommended in the Endocrine Society Clinical Practice Guideline [21], screening laboratories employ a two-tier strategy using liquid chromatography–tandem mass spectrometry in preference to all other methods (e.g., genotyping) to improve the positive predictive value for congenital adrenal hyperplasia by a specific and sensitive second test [21]. This can be more cost-efficient and avoids parental stress. Two-tier screening for CAH [24, 25] has been introduced in the Heidelberg newborn screening by January 2020, after the work-up of this patient. Confirmatory diagnostics would have then been recommended immediately after the initial screening result thereby avoiding the recall sample.

**Case 2:** supposed male, born August 2018; presumptive diagnosis based on clinical phenotype. Step numbers in the text refer to the corresponding step in the flowchart in figure 1.

Due to preeclampsia the infant of healthy, unrelated Caucasian parents was delivered by Caesarean section in the 29 + 2 week of gestation. Postpartum, ambiguous genitals were obvious with anteverted phallus (length 0.5 cm), perineal hypospadia, unfused scrotum but scrotal folds, and undescended gonads (located in the inguinal canal). Associated malformations were anal atresia, malrotation of the intestine, and atrial and ventricular septum defects (step 1). To evaluate the presumptive diagnosis of 46, XY difference of sex development (DSD) the first case conference, organized by the medical coordinator of the centre for rare diseases Heidelberg on day 1 postpartum (pp) together with a paediatric endocrinologist and neonatologist recommended an evaluation according to the guidelines of DSD [26] (step 2) which has been carried out immediately (Step 3).

On day 5 pp, a second case conference was organized by the medical coordinator of the centre for rare diseases Heidelberg. The invited paediatric endocrinologist, geneticist, and the medical evaluator of the steroid hormone laboratory summarised a male karyotype (46, XY), no hypogonadotropic hypogonadism, excretion of premature precursors of the adrenal steroid hormones in the urine, and male serum levels of anti-Mullerian hormone (AMH) indicating testes (step 4). No Müllerian structures were visible in pelvic ultrasound. Although the presumptive diagnosis formulated in step 2 was definitely true positive (step 5) and 46, XY DSD was confirmed, the distinct diagnosis was not yet established. The institution which referred the patient was informed about the true positive result (T^+^) (step 6). Additional molecular genetic analysis, ideally whole exome screening, was recommended (see step 13 below).

The next day, a third case conference was organized by the medical coordinator of the centre for rare diseases Heidelberg. Specialists for paediatric endocrinology, genetics, psychology, paediatric cardiology, paediatric neurology, paediatric surgery, and social work planned the multidisciplinary treatment and care (step 7) [27]. Both parents were informed in detail by the paediatric endocrinologist about the 46, XY DSD and the possibility of ethical counselling, about upcoming cardiac intervention and lifelong monitoring by the cardiologist, necessary operations for inguinal hernia and anal atresia by the paediatric surgeon (step 8). The patient was evaluated by the paediatric neurologist for neurological and motor development, an experienced paediatric psychologist evaluated parent’s coping capacities and supported the family, and a social worker provided information about legal health issues (step 8). Routine monitoring was started (steps 9, 10).

Following the recommendation for additional molecular genetics, the paediatric endocrinologist and the geneticist decided on a trio whole exome sequencing (trio WES) (step 13). After the parents gave their informed consent to innovative genetic diagnostics trio WES was initiated at the age of six month of the patient (step 14). Result became available three months later, reporting a heterozygous de novo frameshift mutation in the *PSMD12* gene. Results were evaluated in a case conference organized with the paediatric endocrinologist, a clinical geneticist, and the geneticist, who performed trio WES. After intensive literature review and matching all available information with the phenotype of the patient they concluded that the detected variant is disease causing (step 15). At the age of nine month, the diagnosis of Stankiewicz-Isidor syndrome (OMIM # 617516) [28] associated with neurodevelopmental disorder, malformation of the heart, kidney, and genitals was made (step 16). Extensive genetic counselling was performed (step 17). The parents were informed about the German patient organization “Intersexuelle Menschen e.V. [29] as well as the Kindernetzwerk e.V.” [30] and were invited to take part in local clinical studies (step 18). The referring institution was informed about the final diagnosis (step 19). Six weeks later, organized by the medical coordinator of the centre for rare diseases Heidelberg, multidisciplinary treatment and care was evaluated according to the checklist (step 20). The patient was followed-up by multidisciplinary outpatient monitoring and required cardiac surgeries were planned (step 21).

**Comment:** Despite considerable progress in our knowledge on the genetic basis of human sexual development only 50% of 46, XY children with DSD will receive a definitive diagnosis [31, 32]. The opportunity to perform trio WES at the age of nine months resulted in the confirmation of a Stankiewicz-Isidor syndrome, a rare condition due to pathologic monoallelic variants in the *PSMD12* gene, first described in 2017 [28]. The phenotype is characterized by variable neurodevelopmental delay and behavioural impairment. Malformations of the heart, kidney, genitals, ears, eyes and skeleton (particularly hands and feet) can also occur.

**Fig. 3:**
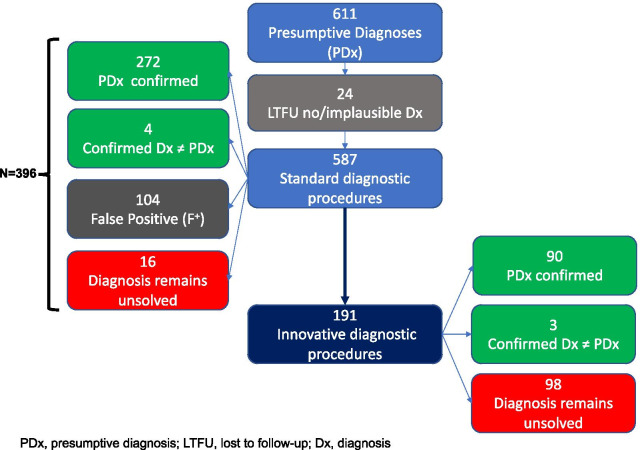
Sample, diagnostic procedures, and diagnostic outcomes of TRANSLATE-NAMSE

From December 2017 to February 2020 611 individuals have been enrolled. Due to lost to follow-up of 24 cases the CPW model could be used to effectively manage 587 individuals with a presumptive diagnose of rare amaemia, endocrinopathy, autoinflammatory disease, primary immune deficiency or inborn error of metabolism. Mean age at first contact was 10.46 years (SD = 13.64, median = 5.8, range 0.0–82.9). As shown in Fig. 3, a definitive diagnosis could be made in 276 from 587 (47%) patients using standard diagnostic procedures according to current consensus guidelines. The presumed diagnosis turned out to be false positive in 104 (17.7%) cases. WES was initiated in 191 (32.5%) patients, of whom 93 (48.7%) received a diagnosis, representing 15.8% of all patients. Overall, diagnosis remained unsolved in 114 cases (19.4%). (Fig. 3 and Table 1). The median duration from first contact to confirmed diagnostic results (including false positive findings) for 88 presumptive diagnoses of the German newborn screening panel (only endocrinopathies and inborn errors of metabolism) after standard diagnostic procedures was 3.5 days (Mean absolute deviation about median, MADmedian = 16.7) which was in line with a previous finding of 4 days from the first report of screening results until the diagnostic confirmation [33]. In contrast for 292 non-screening presumptive diagnoses median time between first contact and confirmed diagnose after standard procedures was 23 days (MADmedian = 36.5). Not surprisingly, median duration from first contact until confirmed diagnosis after standard diagnostic procedures followed by WES was 109 days (MADmedian = 97.5), however, ten times longer than the median of 10.0 days (MADmedian = 29.2) for standard diagnostic procedures alone. Clinical conferences decided that 309 (83.7%) out of 369 patients with a confirmed diagnosis should be treated. Table 2 reports the elements of standardized multiprofessional treatment and care (CPW checklist item 24) which have been executed according to appropriateness for the particular condition (e.g. not all conditions are treated with a diet or require an emergency pass) and patients’/families’ knowledge, skills, and requests (e.g. not all patients want genetic counselling). Overall, the missing value rate in our data base is about 4%. From 104 cases with a false positive presumptive diagnosis information about the result of confirmatory diagnostic procedures was documented for 103 individuals. Except from one out of 114 patients whose diagnosis remained unsolved, information about the diagnostic outcome and/or referral to symptomatic care was documented.

**Table 1:** Numbers of individuals with confirmed presumptive diagnosis, confirmed diagnosis other than presumptive diagnosis, presumptive diagnosis confirmed as false positive, and numbers of individuals in which a diagnosis remained unsolved

Presumptive diagnosis (PDx)	Confirmed diagnosis = PDx	Confirmed diagnosis ≠ PDx*	False positive	Diagnosis remained unsolved	Row total
Rare anaemia					
n	12	0	2	2	16
Row %	75.0	0.0	12.5	12.5	100
Endocrinopathy					
n	121	2	16	6	145
Row %	83.4	1.4	11.0	4.1	100
Autoinflammatory disorder					
n	110	2	49	48	209
Row %	52.6	1.0	23.4	23.0	100
Primary immune deficiency					
n	73	3	30	56	162
Row %	45.1	1.9	18.5	34.6	100
Inborn error of metabolism					
n	46	0	7	2	55
Row %	83.6	0.0	12.7	3.6	100
Column Total	362	7	104	114	587
Row %	61.7	1.2	17.7	19.4	100

^*^Confirmed diagnoses other than PDx: primary ovarian failure (ORPHA 95710), Behcet disease (ORPHA 117), other autoinflammatory disorder (ORPHA 319719), familial mediterranean fever (ORPHA 342), MIRAGE-syndrome (ORPHA 494433), chronic fatigue syndrome, Sneddon-syndrome (ORPHA 820)

**Table 2:** Initiation of standardized multiprofessional treatment and care and information of disease specific resources in 369 individuals with a confirmed diagnosis in TRANSLATE NAMSE

	Executed n (%)	Not appropriate for condition/not necessary* n (%)	Missing data n (%)
Standardized multiprofessionel treatment and care (CPW checklist item 24)			
Referral to further treatment and care	345 (93.5)	0 (0.0)	24 (6.5)
Information diagnosis	346 (93.8)	10 (2.7)	13 (3.5)
Information diet/nutrition	47 (12.7)	308 (83.5)	14 (3.8)
Training for monitoring	215 (58.3)	142 (38.5)	12 (3.3)
Information about medication	217 (58.8)	137 (37.1)	15 (4.1)
Education for behavioural measures	242 (65.6)	112 (30.4)	15 (4.1)
Psychological counselling	144 (39.0)	212 (57.5)	13 (3.5)
Social-legal counselling	100 (27.1)	254 (68.8)	15 (4.1)
Genetic counselling	92 (24.9)	262 (71.0)	15 (4.1)
Delivery emergency pass	50 (13.6)	301 (81.6)	18 (4.9)
Information about disease specific resources (CPW checklist items 26–29)			
Information about research projects	130 (35.2)	226 (61.2)	13 (3.5)
Information about patient registry	150 (40.7)	204 (55.3)	15 (4.1)
Information about patient advocacy group	134 (36.3)	219 (59.3)	16 (4.3)
Information about Alliance for Chronic Rare Diseases	242 (65.6)	104 (28.2)	23 (6.2)

^*^Items may not be appropriate for specific conditions (e.g. emergency pass), individuals may have been already informed or do not wish specific information (e.g. about participation in a registry)

Independent evaluations of the results of TRANSLATE-NAMSE by the Berlin School of Public Health (https://bsph.charite.de/en/) and the Center for Evidence-Based Healthcare, University Hospital Dresden (https://www.uniklinikum-dresden.de/de/das-klinikum/universitaetscentren/zegv/center-for-evidence-based-healthcare/center-for-evidence-based-healthcare) concluded that the implementation of the CPW model was quality-assured, comprehensive, can be effectively used to organise care for individuals with a rare disorder, and that the data model is an appropriate platform for further clinical research. Therefore, results of the newly developed structures and processes allow informed political decisions and have the potential to be included into legally regulated standard care.

## Discussion

Our approach shares some facets with earlier pathway models [15, 34, 35] but is unique combining a generic CPW with available evidence-based guidelines allowing each discipline to comply with their own specific guidelines and granularity of documentation, at the same time creating the essential intersecting set of information necessary for evidence-based clinical decision-making. Pivotal in diagnosis, treatment and care of most rare diseases is that they require expertise from different medical and paramedical subspecialities making a holistic approach compelling [3]. Available disease specific guidelines can be integrated into the generic CPW during its application, but the model also applies to situations where a diagnosis has not yet been made or standard attempts to establish a diagnosis will remain inconclusive. Concrete actions in the different steps of the generic CPW are directed by structured interdisciplinary case conferences, the backbone of the CPW model, enforcing integration of best scientific evidence with clinical experience [19]. Case conferences are of particularly importance also for decisions about innovative genetic diagnostics and to evaluate its results, not only for economic reasons, but a recent estimate suggested that about 25% of patients with rare diseases will not obtain a diagnosis with WGS alone [36]. Even if diagnosis remains unsolved, patients and families should be informed why this is the case, what can be but also what should not be done further on, that novel technologies might bring new results in the future, and finally be referred to symptomatic treatment and care [37]. The CPW model is directly related to individual case management [38] and focusses on the two critical decision nodes. First, a diagnostic result must be established in due time, and second the patient has to be placed into a system of appropriate treatment and care. All case conferences in the two case reports were organised by a medical coordinator, who should be conceived as a moderator of disciplines rather than a case manager. This sounds trivial but deserves consideration, as participants of multiprofessional conferences may come from different departments or even institutions. In our project most conferences and contacts with patients took place in person. Telephone or video conferences can save costs and time, however, will not make face-to-face communication with patients obsolete [37].

The CPW links actions in a complex system, why it has recently been suggested to move away from viewing case management as an intervention which can be evaluated by classical evaluation techniques [38]. However, a CPW makes this system projectable for healthcare providers and healthcare policy, and transparent and understandable for patients and families.

For 369 (62.9%) out of 587 individuals with a presumptive diagnosis a specific diagnosis could be confirmed, thereof 276 (74.8%) using standard diagnostic methods and 93 (25.2%) using innovative diagnostic procedures. Including 104 cases confirmed to be false positive, the overall diagnostic yield was 80.6%. TRANSLATE-NAMSE is the first project in which WES was systematically used to search for diagnoses in patients with rare diseases, why historical data for comparison do not exist. Compared with median diagnostic delays of about five years reported in the literature [36, 39, 40] our process times are much shorter. However, in Germany WES is not easily available for clinical diagnostics so far. The CPW model initially was not designed to be applied on a European level. However, based on the successful implementation and evaluation, it can be a tool in any national plan for rare diseases [41], in particular to European Reference Networks (ERNs), e.g. to ENDO ERN for endocrinological disorders, to METAB ERN for inborn errors of metabolism, and to ERN EuroBloodNet for rare anaemias. Application of the CPW model will present a “virtual” registry which in combination with tissue samples used for the different diagnostic procedures can provide a rich source for research on rare diseases [42].

Patients at the Heidelberg University Hospital were offered participation in an pilot project using a personalized interinstitutional health and patient record giving patients as well as their paediatricians and general practitioners remote access to continuously updated data including the local medical information system [43]. However, a nationwide application will require to solve significant issues of digital inter-operability and data protection. On the other hand, the checklist provided in Fig. 2 can be used as a single electronic form to document the steps of the CPW.

Cost-effectiveness analysis was not in the scope of a health care project as defined by the German Federal Joint Committee. (G-BA). Cost-effectiveness will always depend on the conditions of a particular health care system [44], but the CPW model will allow calculation of costs by pricing each step in a given health system. In our project experts from various institutions and disciplines were refunded according to their contribution by additional appropriations included in the G-BA grant. It will be up to the Federal Joint Committee to decide whether and how to finance new developments and to integrate them into standard treatment and care.

A CPW model for rare diseases can also meet further objectives [15, 45]. First, the flowchart can be used to explain patients and families what they can expect and give them orientation in the sequence of diagnostic steps. Second, health care providers only partially involved or attending specialised treatment and care, like primary physicians can be informed in an easy but comprehensive way. Third, the model can be used to teach novices in the field, combining clinical, scientific, organizational, and economic facets of diagnosis, treatment and care of rare diseases.

## Conclusions

We suggest a generic clinical pathway (CPW) manoeuvring patients with a rare disease from finding the best diagnostic strategy to establish best treatment and care. The CPW model can be combined with available disease specific guidelines but also applies to situations without a guideline. The backbone of the generic CPW is a set of structured multidisciplinary case conferences, projecting and evaluating diagnostic and/or therapeutic steps, thereby enforcing the integration of best scientific evidence with clinical experience. The generic CPW is stated as a flowchart and as a checklist, whereby the latter can be used for parsimonious documentation but also for research. The CPW model is a tool to change the diagnostic odyssey into an organised route, to inform patients and families about the stages of their individual route, to update health care providers only partially involved in treatment and care, and to train novices in the field.

## Data Availability

Not relevant.

## Abbreviations

17-OHP: 17-Hydroxyprogesterone
21-OHD: 21‐Hydroxylase deficiency
21DF: 21-Desoxycortisol
ACHSE: Allianz Chronischer Seltener Erkrankungen (Alliance for Chronic Rare Diseases)
ACTH: Adrenocorticotropic hormone
AMH: Anti-Mullerian hormone
BMG: Bundesministerium für Gesundheit (German Federal Ministry of Health)
CAH: Congenital adrenal hyperplasia
CPW: Clinical pathway
Dx: Diagnosis
ERN: European Reference Network
F^+^: False positive
G-BA: German Federal Joint Committee
i.v.: Intravenous
LTFU: Lost to follow-up
MADmedian: Mean Absolute Deviation about Median
NAMSE: Nationales Aktionsbündnis für Menschen mit seltenen Erkrankungen (National Action League for People with Rare Diseases)
NBS: Newborn screening
OMIM: Online Mendelian Inheritance in Man
PDx: Presumptive diagnosis
p.o.: Per os
pp: Postpartum
T^+^: True positive
WES: Whole exome sequencing
WGS: Whole genome sequencing

## References

[1] Seltene Erkrankungen. https://www.bundesgesundheitsministerium.de/themen/praevention/gesundheitsgefahren/seltene-erkrankungen.html. Accessed 20 Jan 2021.

[2] Ferreira CR (2019). The burden of rare diseases. Am J Med Genet A.

[3] Burgard P (2020). A holistic approach to the patients/ Families with inborn errors of metabolism. J Mother Child.

[4] Council of the European Union (2009). Council Recommendation of 8 June 2009 on an action in the field of rare diseases. Official J Eur Union.

[5] Hoffmann G, Mundlos C, Dötsch J, Hebestreit H. Seltene Erkrankungen in der Pädiatrie–von der Diagnostik und Behandlung einzelner Erkrankungen zum Aufbau von Netzwerkstrukturen. Monatsschrift Kinderheilkunde. 2020:1–13. 10.1007/s00112-020-00978-w.

[6] Union CotE. Council Recommendation of 8 June 2009 on an action in the field of rare diseases. In: Official J Eur Union. 2009. http://eurlex.europa.eu/LexUriServ/LexUriServ.do?uri=OJ:C:2009:151:0007:0010:EN:PDF. Accessed 20.01.2021.

[7] ACHSE. Allianz Chronischer Seltener Erkrankungen (Alliance for Chronic Rare Diseases ). https://www.achse-online.de/de/index.php. Accessed 20 Jan 2021.

[8] NAMSE. Nationales Aktionsbündnis für Menschen mit seltenen Erkrankungen (National Action League for People with Rare Diseases). 2013. https://www.bundesgesundheitsministerium.de/fileadmin/Dateien/3_Downloads/N/NAMSE/Nationaler_Aktionsplan_fuer_Menschen_mit_Seltenen_Erkrankungen_-_Handlungsfelder__Empfehlungen_und_Massnahmenvorschlaege.pdf. Accessed 20 Jan 2021.

[9] National action league for people with rare diseases. National plan of action for people with rare diseases. https://www.namse.de/fileadmin/user_upload/downloads/National_Plan_of_Action.pdf. Accessed 20 Jan 2021.

[10] Gemeinsamer Bundesausschuss. TRANSLATE-NAMSE–Verbesserung der Versorgung von Menschen mit seltenen Erkrankungen durch Umsetzung von im nationalen Aktionsplan (NAMSE) konsentierten Maßnahmen. 2018. https://innovationsfonds.g-ba.de/projekte/neue-versorgungsformen/translate-namse-verbesserung-der-versorgung-von-menschen-mit-seltenen-erkrankungen-durch-umsetzung-von-im-nationalen-aktionsplan-namse-konsentierten-massnahmen.78. Accessed 20 Jan 2021.

[11] Translate Namse. https://translate-namse.charite.de/en/. Accessed 20 Jan 2021.

[12] Grüters-Kieslich A, Burgard P, Berner R, Hoffmann G (2017). Zentren für seltene Erkrankungen. Monatsschrift Kinderheilkunde.

[13] Grasemann C, Matar N, Bauer J, Manka E, Mundlos C, Krude H et al. Development of a structured transition program for adolescents and young adults with a chronic rare disease: Results from the German consortium TRANSLATE NAMSE. Monatsschrift Kinderheilkunde. 2020:1–9. 10.1007/s00112-020-00929-5.

[14] Rotter T, Kinsman L, James E, Machotta A, Gothe H, Willis J (2010). Clinical pathways: effects on professional practice, patient outcomes, length of stay and hospital costs. Cochrane Database Syst Rev.

[15] Campbell H, Hotchkiss R, Bradshaw N, Porteous M (1998). Integrated care pathways. BMJ.

[16] Demirdas S, van Kessel IN, Korndewal MJ, Hollak CE, Meutgeert H, Klaren A (2013). Clinical pathways for inborn errors of metabolism: warranted and feasible. Orphanet J Rare Dis.

[17] Léger J, Olivieri A, Donaldson M, Torresani T, Krude H, Van Vliet G (2014). European Society for Paediatric Endocrinology consensus guidelines on screening, diagnosis, and management of congenital hypothyroidism. Hormone Res Paediatr.

[18] Burgard P, Rupp K, Lindner M, Haege G, Rigter T, Weinreich SS (2012). Newborn screening programmes in Europe; arguments and efforts regarding harmonization. Part 2. From screening laboratory results to treatment, follow-up and quality assurance. J Inherit Metab Dis.

[19] Djulbegovic B, Guyatt GH (2017). Progress in evidence-based medicine: a quarter century on. Lancet.

[20] Gemeinsamer Bundesausschuss. Richtlinie des Gemeinsamen Bundesausschusses über die Früherkennung von Krankheiten bei Kindern (Kinder-Richtlinie)[Stand: 19. Oktober 2017]. In: Published in Bundesanzeiger AT. 2017. https://www.g-ba.de/richtlinien/15/. Accessed 18 Jan 2021.

[21] Speiser PW, Arlt W, Auchus RJ, Baskin LS, Conway GS, Merke DP (2018). Congenital adrenal hyperplasia due to steroid 21-hydroxylase deficiency: an endocrine society clinical practice guideline. J Clin Endocrinol Metab.

[22] GLANDULA Netzwerk Hypophysen- und Nebennierenerkrankungen e.V. https://www.glandula-online.de/. Accessed 20 Jan 2021.

[23] AQUAPE/AGS. https://buster.zibmt.uni-ulm.de/projekte/AGS/. Accessed 20 Jan 2021.

[24] Janzen N, Peter M, Sander S, Steuerwald U, Terhardt M, Holtkamp U (2007). Newborn screening for congenital adrenal hyperplasia: additional steroid profile using liquid chromatography-tandem mass spectrometry. J Clin Endocrinol Metab.

[25] Janzen N, Riepe FG, Peter M, Sander S, Steuerwald U, Korsch E (2012). Neonatal screening: identification of children with 11β-hydroxylase deficiency by second-tier testing. Hormone Research in Paediatrics.

[26] Arbeitsgemeinschaft der Wissenschaftlichen Medizinischen Fachgesellschaften (AWMF). S2k-Leitlinie „Varianten der Geschlechtsentwicklung (AWMF-Registernummer 174/001) 2016. https://www.awmf.org/leitlinien/detail/ll/174-001.html. Accessed 20 Jan 2021.

[27] Lee PA, Houk CP, Ahmed SF, Hughes IA (2006). Consensus statement on management of intersex disorders. Int Consensus Conf Intersex Pediatr.

[28] Küry S, Besnard T, Ebstein F, Khan TN, Gambin T, Douglas J (2017). De novo disruption of the proteasome regulatory subunit PSMD12 causes a syndromic neurodevelopmental disorder. Am J Hum Genet.

[29] Intersexuelle Menschen. https://im-ev.de/. Accessed 20.01.2021.

[30] Kindernetzwerk. https://www.kindernetzwerk.de/de/. Accessed 20 Jan 2021.

[31] Ahmed SF, Cheng A, Dovey L, Hawkins JR, Martin H, Rowland J (2000). Phenotypic features, androgen receptor binding, and mutational analysis in 278 clinical cases reported as androgen insensitivity syndrome. J Clin Endocrinol Metab.

[32] Morel Y, Rey R, Teinturier C, Nicolino M, Michel-Calemard L, Mowszowicz I (2002). Aetiological diagnosis of male sex ambiguity: a collaborative study. Eur J Pediatr.

[33] Lindner M, Gramer G, Haege G, Fang-Hoffmann J, Schwab KO, Tacke U (2011). Efficacy and outcome of expanded newborn screening for metabolic diseases-Report of 10 years from South-West Germany. Orphanet J Rare Dis.

[34] Coffey RJ, Richards JS, Remmert CS, LeRoy SS, Schoville RR, Baldwin PJ (1992). An introduction to critical paths. Qual Manag Health Care.

[35] Kitchiner D, Bundred P (1996). Integrated care pathways. Arch Dis Child.

[36] Wu AC, McMahon P, Lu C (2020). Ending the diagnostic odyssey-is whole-genome sequencing the answer?. JAMA Pediatr.

[37] Carmichael N, Tsipis J, Windmueller G, Mandel L, Estrella E (2015). "Is it going to hurt?": the impact of the diagnostic odyssey on children and their families. J Genet Couns.

[38] Lambert AS, Legrand C, Cès S, Van Durme T, Macq J (2019). Evaluating case management as a complex intervention: Lessons for the future. PLoS ONE.

[39] Blöß S, Klemann C, Rother A-K, Mehmecke S, Schumacher U, Mücke U (2017). Diagnostic needs for rare diseases and shared prediagnostic phenomena: results of a German-wide expert Delphi survey. PLoS ONE.

[40] Sawyer S, Hartley T, Dyment D, Beaulieu C, Schwartzentruber J, Smith A (2016). Utility of whole-exome sequencing for those near the end of the diagnostic odyssey: time to address gaps in care. Clin Genet.

[41] Commission E. Non-communicable diseases; Rare diseases. https://ec.europa.eu/health/non_communicable_diseases/rare_diseases_en. Accessed 22 Sept 2021.

[42] Garcia M, Downs J, Russell A, Wang W (2018). Impact of biobanks on research outcomes in rare diseases: a systematic review. Orphanet J Rare Dis.

[43] Die PEPA – eine persönliche, einrichtungsübergreifende Gesundheits- und Patientenakte am Universitätsklinikum Heidelberg und in der Metropolregion Rhein-Neckar. https://pepa.eu/. Accessed 26 May 2021.

[44] Pfeil J, Listl S, Hoffmann GF, Kölker S, Lindner M, Burgard P (2013). Newborn screening by tandem mass spectrometry for glutaric aciduria type 1: a cost-effectiveness analysis. Orphanet J Rare Dis.

[45] European Pathway Association. http://e-p-a.org/. Accessed 24 Jan 2021.

